# CHEK1 variant is a risk factor for premature ovarian insufficiency by mis- regulating metabolism and inflammation-related genes

**DOI:** 10.1186/s40246-025-00774-1

**Published:** 2025-06-18

**Authors:** Jianying Guo, Yali Fan, Zifan Song, Lin Li, Meng Fu

**Affiliations:** 1 State Key Laboratory of Female Fertility Promotion, Center for Reproductive Medicine, Department of Obstetrics and Gynecology, Peking University Third Hospital, Beijing, 100191 China; 2 National Clinical Research Center for Obstetrics and Gynecology, Beijing, 100191 China; 3 Key Laboratory of Assisted Reproduction (Peking University), Ministry of Education, Beijing, 100191 China; 4 Beijing Key Laboratory of Reproductive Endocrinology and Assisted Reproductive Technology, Beijing, 100191 China; 5 Central Laboratory, Beijing Obstetrics and Gynecology Hospital, Capital Medical University, Beijing Maternal and Child Health Care Hospital. Dongcheng, Beijing, 100006 China; 6 Department of Reproductive Medicine, Haidian District Maternal and Child Health Care Hospital, No.53 Suzhou Street, Haidian, Beijing, 100080 China

**Keywords:** Premature ovarian insufficiency, CHEK1, Variant, Assisted reproduction, Metabolism

## Abstract

**Background:**

Premature ovarian insufficiency (POI) is affecting approximately 1% of females and increasingly contributing to female infertility. The etiology of POI is heterogeneous. CHEK1, a critical component of the DNA damage and replication stress response, has recently been linked to female reproductive biology.

**Results:**

We identified a CHEK1 variant c.77C > G; p.A26G in a POI patient through whole-exome sequencing. Protein structure prediction and pathogenicity analysis suggested that the CHEK1 A26G variant may affect protein stability. RNA sequencing results of 293FT cells overexpressing wild-type and A26G CHEK1 revealed altered expression and alternative splicing of genes involved in metabolism and inflammation.

**Conclusion:**

CHEK1 may be involved in ovarian aging and the A26G variant may increase susceptibility to POI.

**Supplementary Information:**

The online version contains supplementary material available at 10.1186/s40246-025-00774-1.

## Background

Premature ovarian insufficiency (POI) is characterized by deficient ovarian sex hormones and decreased ovarian reserve, affecting approximately 1% females under the age of 40 [1]. As a growing number of women desiring conception beyond the age of 30, POI is becoming a major cause of female infertility. POI is highly heterogeneous and the etiology of POI is mostly underdetermined. Numbers of chromosomal abnormality and genetic mutations located on autosomal and sex chromosomal evolved in gonadal development, meiosis, DNA repair, metabolism, immune response and hormone secretion were proved to be genetic causes of POI, while non-genetic cause of POI include immune and metabolic disorders, infections, environmental factors and iatrogenic reasons. Recently, submicroscopic copy number variations (CNVs) and whole-exome sequencing (WES) are being used for investigating novel POI related genes or regions and have greatly expanded understanding of the POI pathogenesis [2].

CHEK1 is a major component of the DNA damage and replication stress responses. Recently, The CHEK1 protein has been proved to correlate with female reproductive biology. CHEK1 and other genes such as CDK1, FANCI, MMC4 were downregulated in theca interna during atresia [3], indicating the vital role of DNA damage repair and cell cycle related proteins in ovarian aging. Through analysis of a large-scale genome-wide association (GWAS) data, several novel polygenic causes of POI including CHEK1 and CHEK2 were revealed. Gain of function of CHEK1 established a larger ovarian reserve at birth, resulting in delayed reproductive ageing in mouse model [4]. Inhibition of CHEK1 could cause accumulation of DNA damage and increase apoptosis in ovarian proliferating somatic cells with no harm to primordial oocytes [5]. Besides, inhibition of CHEK1 during chemotherapy showed a protective effect on human oocyte from apoptosis and enhanced tumor chemotherapeutic efficacy through the CHEK-TAp63a pathway [6].

Our previous report found that the maternal mutation in CHEK1 leads to mitotic arrest in human zygotes thus resulting in female infertility [7]. Building on these findings, the present study aimed to investigate the potential impact of CHEK1 on ovarian function. Herein, we found a POI patient with CHEK1 c.77C > G; p.A26G (A26G for short) variant through WES. Structural modeling and pathogenicity prediction suggested that the CHEK1 A26G variant may impair protein stability. Under identical experimental conditions, overexpression of the mutant CHEK1 in 293FT cells showed a trend of reduced protein expression compared to the wild-type. Furthermore, transcriptome analysis revealed that differentially expressed genes between the mutant and wild-type overexpression groups were enriched in metabolic and inflammatory related pathways.

## Methods

### Whole exome sequencing

5 ml of Peripheral Blood was collected from the patient with POI or control for DNA library construction using Agilent SureSelect Human All Exon V6 kit (Agilent Technologies, Santa Clara, CA, USA). CHEK1 c.77C > G heterogenous variant was validated by PCR and sanger sequencing.

### RNA sequencing and data analysis

RNA extraction and sequencing was carried out by BerryGenomics Co., Ltd. (Beijing, China). Differential gene expression was performed using the DESeq2 package of R. Genes with P-values < 0.05 and FDR values < 0.05 were considered differentially expressed. The DEGs were further analyzed by AmiGo (https://amigo.geneontology.org/amigo) for gene ontology classification. Alternative splicing events between WT and A26G CHEK1 overexpressed cells were analyzed using rMATS version 3.2.2 software with hg19 refGene annotation. Five types of alternative splicing events were annotated based on Ensemble genes and were tested for an inclusion-level difference of > 5%. Events with a false discovery rate (FDR) of less than 0.05 were identified as differentially expressed alternative splicing events. Additional methods and primer sequences can be found in Supplementary Methods (Additional file 2) and Supplementary Table 1 (Additional file 6).

## Results

### Identification of CHEK1 c.77C > G; p.A26G as a novel variant in POI patient

**Fig. 1:**
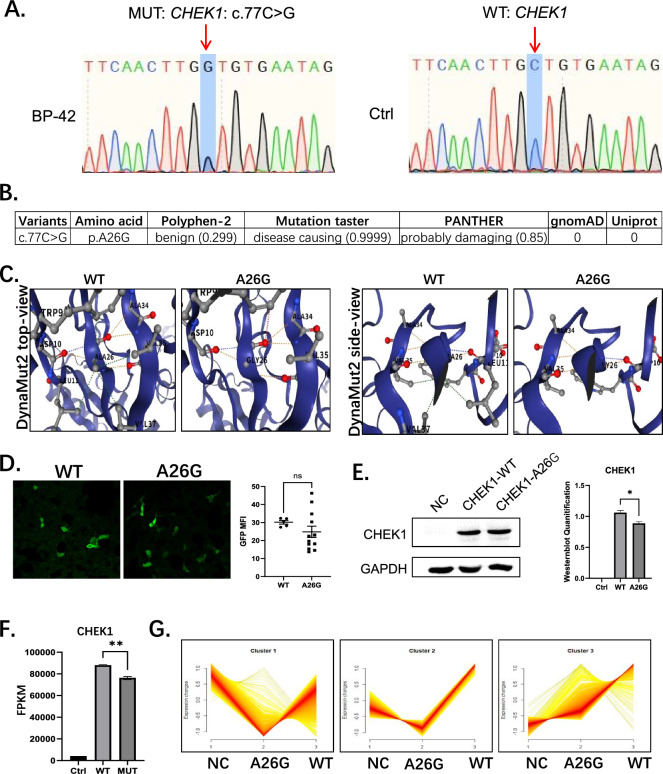
Identification of CHEK1 c.77C > G; p.A26G as a novel variant in POI patient. **A** A Chinese POI patient with CHEK1 c.77C > G; p.A26G variant validated by sanger sequencing. **B** In silico analysis of CHEK1 variant by Polyphen-2 (http://genetics.bwh.harvard.edu/pph2/), Mutation Taster (http://www.mutationtaster.org/), PANTHER (http://www.pantherdb.org/tools/csnpScoreForm.jsp), gnomAD (https://gnomad.broadinstitute.org/) and Uniport. **C** Structures prediction of wild type (WT) and A26G variant by DynaMut2, presented from the top-view (left) and side-view (right). The blue lines represent the secondary β-sheet structure of the protein, and atoms are displayed in ball-and-stick model. Dashed lines indicate potential interactions (such as hydrogen bonds). **D** Immunostaining and quantification of HA-Tag showing the location and intensity of ectopic overexpressed WT and A26G CHEK1. Lines and columns are means ± SEM. MFI, mean fluorescent intensity. ns, not significant by t-student test. **E** Western blot and quantification showing the overexpression of WT and A26G CHEK1. N = 3 replicates. Lines and columns are means ± SEM. *p < 0.05 by t-student test. **F** Bar plot showing the FPKM of CHEK1 transcript by RNA-sequencing in NC, WT and A26G CHEK1 overexpressed groups. N = 3 replicates. Lines and columns are means ± SEM. **p < 0.01 by t-student test. **G** Mfuzz gene clustering analysis for NC, WT and A26G CHEK1 overexpressed groups. x axis, different groups as labeled; y axis, gene expression level in normalized count value. Three distinct cluster patterns were classified by Mfuzz (R package)

The clinical diagnose of POI is women under 40 years of age with oligomenorrhea/amenorrhea > 4 months; FSH (follicle-stimulating hormone) levels > 25 U/L twice (more than 4 weeks apart). We identified a 34-year-old POI patient with FSH levels of 43.8 U/L and 115.76 U/L, and an AMH level of < 0.06, indicating insufficient ovarian function. Whole-exome sequencing, performed with informed consent, revealed a heterozygous variant c.77C > G; p.A26G in the CHEK1 gene (Additional file 4: Supplementary Table 2). The c.77C > G variant generates a missense protein variant, with p.A26G (A26G for short), and we validated the variant by Sanger Sequencing (Fig. 1A).

Since CHEK1 is crucial for cell cycle regulation and reproductive lifespan [4], we examined CHEK1 interaction proteins using the BioGRID (thebiogrid.org) database. PANTHER Overrepresentation Test indicates that most of the interactors’ function enriched in P53 pathway, EGF receptor signaling pathway and gonadotropin-releasing hormone receptor pathway (Additional file 1: Supplementary Figures Fig. S1A). GO (Gene Ontology) analysis of the interactors also showed an enrichment in cell cycle and DNA metabolism regulation related terms (Additional file 1: Supplementary Figures Fig. S1B).

According to Varsome dabase, CHEK1 is currently linked to the phenotype of oocyte, zygote and embryo arrest, and the inheritance mode is Autosomal Dominant (AD) (Additional file 5: Supplementary Table 3). For the CHEK1 c.77C > G (p.Ala26Gly) variant found in our patient, in silico predictions from MutationTaster and PANTHER suggest a potentially damaging effect, while PolyPhen-2 classifies it as benign (Fig. 1B). The variant is classified as Variant of Uncertain Significance (VUS) according to ACMG guidelines as interpreted by VarSome (PM2 + PP3-BP1). The automated criteria include both pathogenic and benign supporting evidence, with 4 tools predict a pathogenic effect, 14 tools are of uncertain and 8 are benign/moderate effect (Additional file 5 Supplementary Table 3). In silico predictions for the CHEK1 A26G variant were inconclusive, highlighting the importance of combining computational and experimental approaches.

The protein kinase domain of the CHEK1 is 9–265 in amino acid, followed by a disordered linker peptide with multiple post-translational modification sites, then end up with an autoinhibitory region from 391–476 aa. Our variant, A26G, located at the 19–28 beta strand in protein kinase domain, between two ATP binding sites 15–23 and 38–38 (Additional file 1: Supplementary Figures Fig. S2A). The Ala located at animo acid 26 in human CHEK1 is conserved across species. We then conducted structural analysis based on the AlphaFold-predicted model. The mutant structure was subjected to DynaMut2 analysis, which indicated a ΔΔG value of -0.98 kcal/mol, suggesting a destabilizing effect on protein stability. The residue A26 of CHEK1 is embedded within a well-structured β-strand and engages in backbone hydrogen bonding essential for β-sheet stability. Substitution of alanine with glycine (A26G) introduces increased flexibility and may disrupt these key hydrogen bonds, potentially compromising the local secondary structure and affecting overall protein stability and function, indicating a potentially pathogenic effect (Fig. 1C). Moreover, AlphaMissense Pathogenicity Heatmap shows the variant A26G is marked with a score of 0.754, located in a relatively red area, suggesting a potentially high pathogenicity (Additional file 1: Supplementary Figures Fig. S2B).

In summary, multiple in silico tools and structural predictions suggest a potentially pathogenic effect of this variant. Thus, we hypothesized that the A26G variant may contribute to the patient's POI phenotype.

### Overexpress WT and A26G CHEK1 in 293FT cells

To test whether the A26G variant affects the CHEK1 function, we cloned human wild type (WT) CHEK1 and generated the same CHEK1 A26G variant (MUT) as found in the POI patient with HA tags for identification. 293FT cells were chosen for in vitro overexpressing the constructs due to their high transfection efficiency and suitability for transcriptomic studies (Additional file 1: Supplementary Figures Fig. S2C). Immunofluorescence showed similar localization patterns for both proteins, with strong nuclear signals and weaker cytoplasmic signals. The mean fluorescent intensity (MFI) of GFP stained Tag showed a decreasing trend in MUT group compared to WT, but is not statistically significant (Fig. 1D). Western blot analysis showed the variant does not affect the size of CHEK1, and the CHEK1 expression level relative to GAPDH was lower in MUT group (Fig. 1E) (Additional file 3: Supplementary Material). We then compared the percentages of dividing cells between the two groups. Cells in interphase or mitotic phases were identified by nuclear morphology. The percentage of mitotic phase was 0.049 in un-transfected 293FT cells, while after transfection, mitotic phase rate decreased to 0.026 and 0.025 in WT and MUT group, respectively (Additional file 1: Supplementary Figures Fig. S2D and S2E), indicating both proteins can arrest unperturbed cell cycle. We next repeated the ectopic expression of the WT and A26G variant in 293FT cells for total mRNA transcriptome analysis. RNA-Seq data revealed an elevation of CHEK1 transcript expression in both WT and MUT group, with the FPKM fold change of MUT compared to WT is 0.87, and the adjusted p-value is 0.0011(Fig. 1F). This result is consistent with our Western blot results. Transcriptome analysis indicates 283 DEGs changed between WT and MUT transfected groups (Additional file 1: Supplementary Figures Fig. S3A). The DEGs can be classified to three clusters, and interestingly, DEGs enriched in A26G always showed down regulation trends compared to WT CHEK1 overexpression group (Fig. 1G). These results indicate compared to WT, transfection of the A26G variant in 293FT cells resulted in a lower level of CHEK1 overexpression under the same conditions, causing a different transcriptome change.

### CHEK1 A26G alters expression of genes involved in metabolism and immune response

**Fig. 2:**
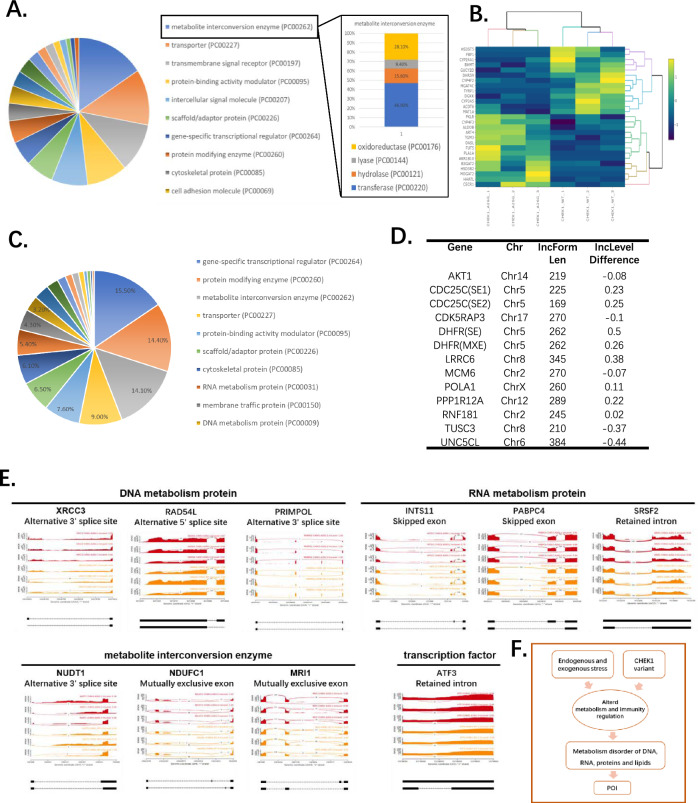
Altered expression and splicing of metabolism and transcriptional regulation related genes between CHEK1 A26G and WT. **A** Pie chart showing DEGs classified by Panther protein classes and subclasses. **B** Heatmap showing the expression level of metabolite interconversion enzymes in WT and A26G CHEK1 overexpression 293FT cells. **C** Pie chart showing AS genes classified by Panther protein classes. **D** AS genes that are also direct interactors of CHEK1 protein. **E** Sashimi plot showing key AS genes functioned as DNA metabolism protein, RNA metabolism protein, metabolite interconversion enzyme or transcription factor. Skipped exon (SE), mutually exclusive exon (MEX), alternative 5′ splice site (A5SS), alternative 3′ splice site (A3SS), and retained intron (RI). **F** Schematic representation shows the model of CHEK1 variant as a risk factor of primary ovarian insufficiency

To examine whether the A26G mutant may alter the function of CHEK1 protein, we looked in detail on the DEGs between A26G and WT CHEK1 overexpressed groups. Heatmap of the 283 DEGs revealed distinct expression pattern in subset of genes in WT versus mutant CHEK1 overexpressed cells (Additional file 1: Supplementary Figures Fig. S3B). Among these DEGs, 155 genes were upregulated and 128 genes were downregulated in A26G CHEK1 variant (Additional file 1: Supplementary Figures Fig. S3C). Further Panther pie chart (https://pantherdb.org/) of DEGs classification based on protein class indicated that the topmost three classes were metabolite interconversion enzyme (32 DEGs), transporter (25 DEGs) and transmembrane signal receptor (22 DEGs). Within the metabolite interconversion enzyme class, the main subclasses included transferases (46.90%), oxidoreductases (28.10%), hydrolases (15.60%), and lyases (9.40%) (Fig. 2A). KEGG pathway analysis further indicated significant enrichment in pathways related to metabolism and ligand-receptor interactions (Additional file 1: Supplementary Figures Fig. S3D). Gene Ontology analysis confirmed that the differentially expressed genes were enriched in ion/lipid transport by biological process, binding/channel activity by molecular function and plasma membrane/cell periphery by cellular component (Additional file 1: Supplementary Figures Fig. S3E). The heatmap of metabolite interconversion enzymes indicated a marked difference between groups, suggesting altered metabolic regulation due to the A26G mutation (Fig. 2B).

Notably, IFIT1 and IFIT2—two RNA-binding proteins involved in antiviral responses and apoptosis—were among the most significantly upregulated DEGs (Additional file 1: Supplementary Figures Fig. S3F), and these results were confirmed by quantitative-PCR (Additional file 1: Supplementary Figures Fig. S3G). Collectively, transcriptome analysis indicated that the A26G variant induces alterations in cellular metabolism and immune-related pathways compared to WT CHEK1.

### CHEK1 A26G cause alternative splicing of genes regulating cellular stress response

We next analyzed the alternative splicing (AS) events with WT and A26G CHEK1 expressing cells. A total of 366 genes were alternatively spliced significantly, with 56 genes exhibiting multiple AS events (FDR < 0.05). GO analysis of the 366 alternative splicing genes showed enrichments on nitrogen compound metabolic process, binding and catalytic activity (Additional file 1: Supplementary Figures Fig. S3H). Panther pie chart classified these AS genes to transcriptional regulation (15.5%), protein-modifying enzyme (14.4%), and metabolite interconversion enzyme (14.1%) (Fig. 2C). Among those, 11 AS genes (AKT1, CDC25C, CDK5RAP3, DHFR, LRRC6, MCM6, POLA1, PPP1R12A, RNF181, TUSC3, UNC5CL) were also direct interactors of CHEK1 protein. Many of those genes were key regulators for responding extracellular signals, thus playing important roles on several major cellular processes such as metabolism, proliferation, cell survival, growth, angiogenesis and apoptosis (Fig. 2D). We also noticed many AS genes were functioned as metabolic regulators of energy, DNA and RNA. Such as metabolite interconversion enzyme (NUDT1, NDUFC1, MRI1), DNA metabolism protein (XRCC3, RAD54L, PRIMPOL), RNA metabolism protein (INTS11, PABPC4, SRSF2) and transcription factor (ATF3) (Fig. 2E).

These findings raise the possibility that the CHEK1 A26G variant contributes to altered cellular metabolism through both changes in gene expression and alternative splicing (AS) events. A26G mutant CHEK1 overexpressed cells showed several AS genes on major regulation pathway such as AKT1 and ATF3, which were key regulators of modulating biological processes of cell growth, causing distinct responses to extracellular stimuli. The resulting dysregulation of metabolic and immune pathways may contribute to a sustained inflammatory microenvironment, potentially implicating this variant in the pathogenesis of POI (Fig. 2F).

## Discussion

POI is a major cause of female infertility, with genetic factors implicated in approximately 20–30% of POI cases. Recent advances using WES have identified several novel genes and variants associated with POI or reproductive lifespan, including EIF4ENIF1, AMHR2, NOBOX, DAZL, KHDRBS1, CCDC155, RNPC3, SPO16, TP63, PRDM9 and ANKRD31. Among these genes, variants related to DNA repair, meiosis and mitosis have been linked to the occurrence of POI and contributed to 37.4% of all genetic causes of POI [8]. Those findings established the vital role of cell cycle regulation genes in the pathogenesis of POI. A recent study identifying novel genes associated with menopause timing highlights the significance of genes involved in DNA damage repair. Menopause is frequently linked to the accumulation of DNA damage and a decline in repair efficiency. Notably, transgenic mice with an extra copy of CHEK1 exhibit a higher egg count and delayed onset of menopause, suggesting that CHEK1 plays a novel role in extending reproductive lifespan [9].

In this study, we identified a heterozygous *CHEK1* A26G variant in a patient diagnosed with POI. Functional experiments in 293FT cells suggest that the mutant protein may have a diminished regulatory capacity compared to the wild-type form, and may affect gene expression and alternative splicing patterns related to cellular metabolism and immune responses. These findings raise the possibility that the A26G variant could influence the ovarian microenvironment, either by impairing genomic surveillance or by modulating downstream pathways involved in metabolic homeostasis. These results were in consistent with a previous study indicating a variant of CHEK1 with a truncated N-terminal region can inhibit its kinase activity, highlighting the importance of the N-terminal ATP-binding domain in regulating CHEK1 function. Our previous study [7] demonstrated that gain-of-function mutations of CHEK1 lead to the failure of zygote cleavage and subsequent female infertility. Together with this finding, our research highlighted the crucial role of CHEK1 dosage in female reproductive function, both on oocyte/zygote and on ovarian somatic cells forming the microenvironment [10].

This study has several limitations. First, the CHEK1 c.77C > G (p.A26G) variant was identified in a single POI patient, and while it emerged as the most likely candidate after stringent variant filtering, the possibility of other undetected or uncharacterized variants contributing to the POI phenotype cannot be ruled out. Therefore, the potential association between the CHEK1 A26G variant and POI needs further validation in larger cohorts to assess its recurrence and pathogenicity. Second, while 293FT cells served as a tractable model for transcriptomic and alternative splicing analysis, they do not fully represent ovarian cells. Given the observed alterations in genes like AKT1 and ATF3, which are involved in ovarian function, the use of ovarian-derived cell models would better reflect the physiological conditions and should be prioritized in future studies. Finally, while this study provides preliminary insights into the potential role of the CHEK1 in POI, it is based on a single case and in vitro data. Further research using larger patient cohorts and ovarian-specific models is required to validate these findings and confirm the broader relevance of CHEK1 in POI pathogenesis.

## Conclusion

In conclusion, our findings suggest that the CHEK1 A26G variant may influence ovarian function by destabilizing the CHEK1 protein structure, leading to altered gene expression and alternative splicing in pathways related to metabolism and immune responses. The variant appears to affect cellular responses to DNA damage and stress, potentially contributing to the dysregulated microenvironment observed in POI. Although these results were demonstrated in 293FT cells, further studies using ovarian-derived models and larger patient cohorts are necessary to validate these findings and investigate the broader pathogenic role of CHEK1 in POI. This study highlights the potential involvement of CHEK1 in ovarian function regulation and provides a foundation for future research focused on understanding the genetic basis of POI and developing early diagnostic strategies for intervention.

## Supplementary Information

**Additional file 1**. Supplementary Figures.

**Additional file 2**. Supplementary Methods.

**Additional file 3**. Supplementary Material. Raw Western Blotting.

**Additional file 4**. Supplementary Table 2. BP42-filtered variants.

**Additional file 5**. Supplementary Table 3. Varsome prediction.

**Additional file 6**. Supplemental Table 1. List of primer sequences used in this study.

## Data Availability

No datasets were generated or analysed during the current study.
