# Supplementary material for: CHEK1 variant is a risk factor for premature ovarian insufficiency by mis- regulating metabolism and inflammation-related genes
Source: Hum Genomics. 2025 Jun 18;19:67. doi: 10.1186/s40246-025-00774-1 (PMC12178055; doi:10.1186/s40246-025-00774-1)

Fig S1. CHEK1 interactors enriched in GO term of cell cycle and DNA metabolic related processes

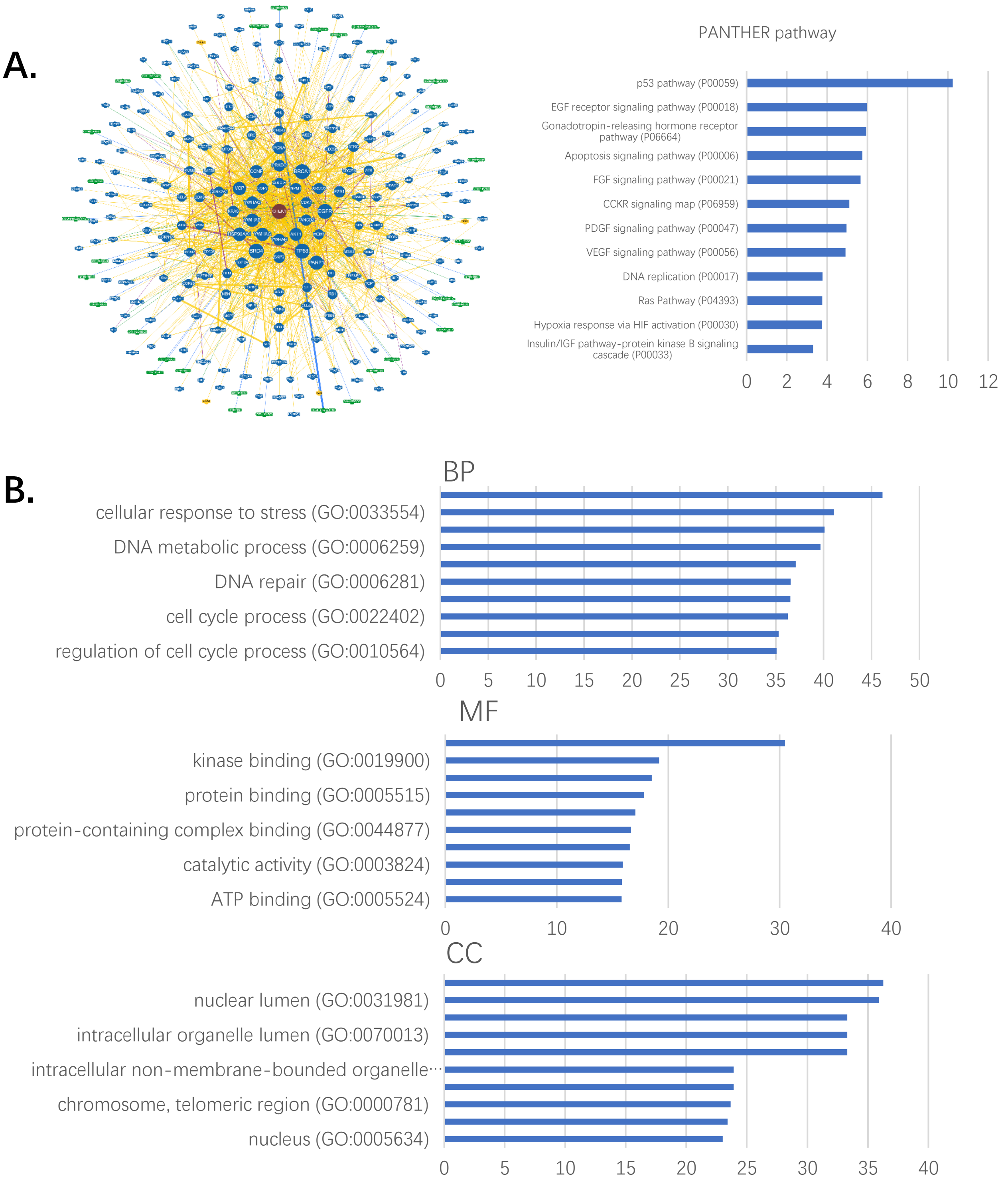

Fig S2. CHEK1 structure and overexpression in 293FT cells

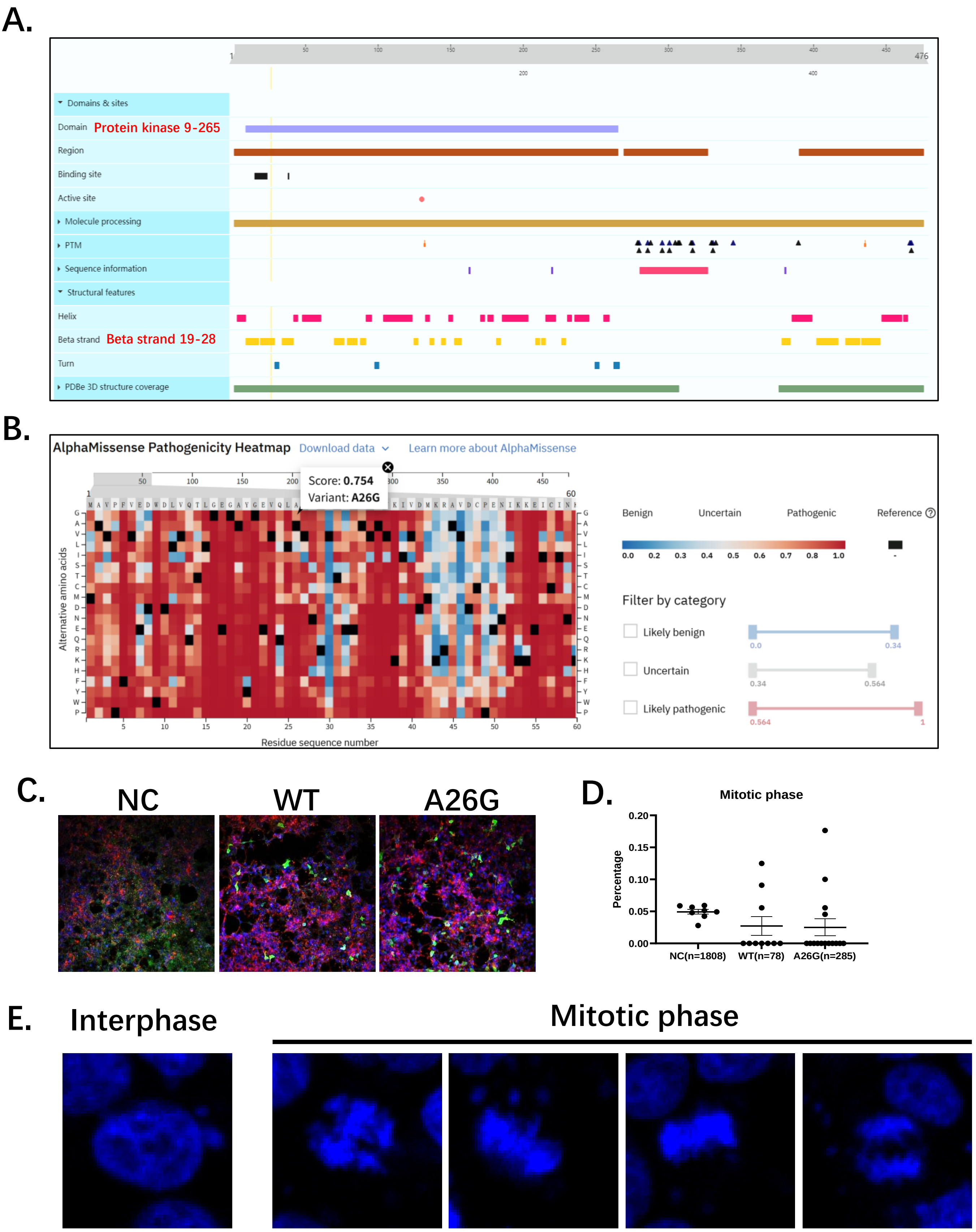

Fig S3. Comparison of transcriptome and splicing change between CHEK1 A26G and WT

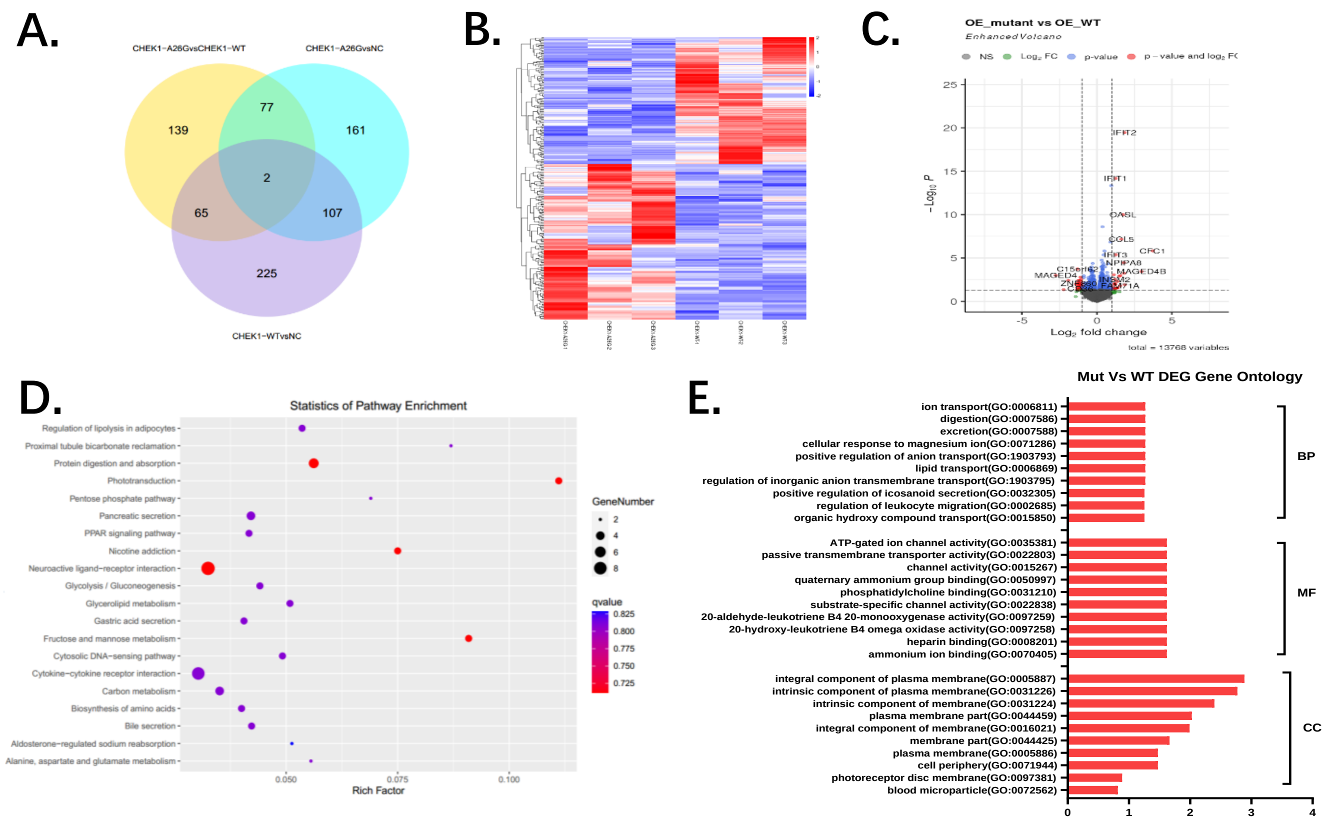

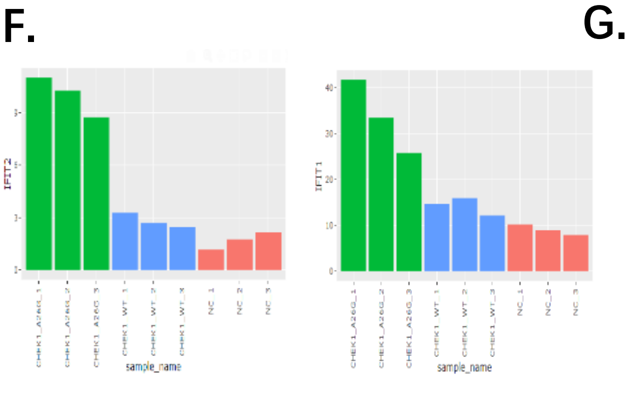

G.

	NC	WT	Mut
IFIT2	1	1.12879	3.265735
SD	0.348053	0.115992	0.159999

	NC	WT	Mut
IFIT1	1	0.771756	2.624213
SD	0.562002	0.177607	0.397196

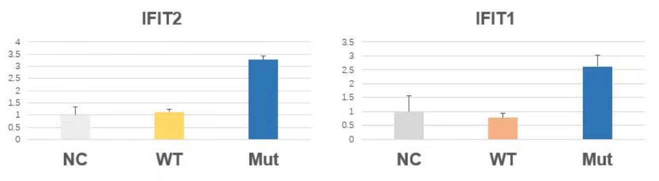

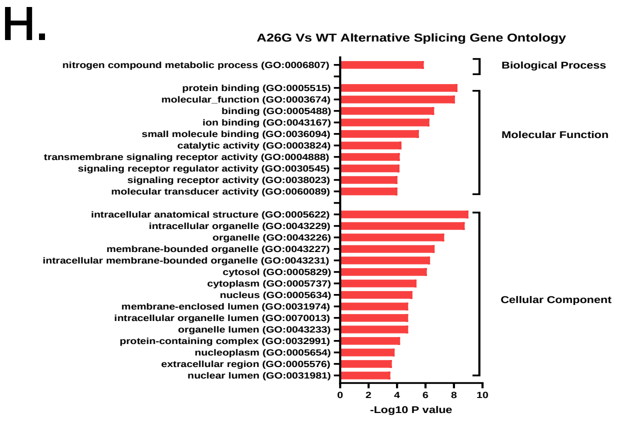

Supplement: Supplementary file 1 — Additional file 1. Supplementary Figures. [file 40246_2025_774_MOESM1_ESM.pdf]
