# Supplementary material for: CHEK1 variant is a risk factor for premature ovarian insufficiency by mis- regulating metabolism and inflammation-related genes
Source: Hum Genomics. 2025 Jun 18;19:67. doi: 10.1186/s40246-025-00774-1 (PMC12178055; doi:10.1186/s40246-025-00774-1)
Supplement: Supplementary file 2 — Additional file 2. Supplementary Methods. [file 40246_2025_774_MOESM2_ESM.docx]

**Supplementary Material and Method**

**Plasmid construction**

The full length human CHEK1 coding sequence (NM_001114121) was amplified by PCR from cDNA of 293FT cells and cloned into pcDNA3.1 vector with HA tag for ectopic expression. CHEK1 c.77C>G variant plasmid was cloned by overlapping PCR of the WT plasmid for importing the point mutation.

**Cell culture and plasmid transfection**

293FT cells were cultured in the medium consisting of DMEM (Gibco) with 15% FBS (HyClone), 1× Nonessential Amino Acids (Invitrogen), 1 mM GlutaMAX (Invitrogen) and 1% penicillin-streptomycin (Gibco). Cells were passaged for approximately 2-3 days when reached a confluency about 90% using 0.05% Trypsin-EDTA (Gibco) at 1:10 ratio.

For ectopic overexpression of WT and A26G CHEK, 293FT cells were seeded on six-well plate at 30% confluency and transfected using Lipofectamine 3000 (Invitrogen) following the manufacture’s instruction. Empty plasmid backbone was transfected in parallel as negative control. Cells were harvested after 48hrs after transfection for further characterization.

**Immunofluorescence**

Transfected 293FT cells were fixed with 4% paraformaldehyde (PFA) at room temperature (RT) for 15 minutes, rinsed twice by PBS, followed by permeabilized at RT for 10 min in 0.3% Triton-X-100 and 0.1% glycine in PBS. Primary antibody targeting HA tag (Proteintech, 51064-2-AP) was diluted in blocking buffer consisting of PBS plus 10% FBS, 1% Tween-20 as desired dilution, and incubated at 4°C overnight. Cells were then washed in PBS twice and incubated in secondary antibody together with F-ACTIN (Abcam, ab176759, for cell shape) and DAPI (Sigma-Aldrich, D9542, for nuclei) in blocking buffer for 1h at RT. After secondary incubation, cells were washed twice and maintained in PBS for confocal imaging.

**Quantitative PCR**

Total RNA of transfected 293FT cells were extracted with TRIZOL (Invitrogen). Reverse transcription was conducted with Superscript II (Invitrogen) starting from 1μg of total RNA. Q-PCR reactions were performed using GoTaq Master Mix (Promega). The relative expression level of each gene was normalized against GAPDH. Statistic represents Student’s t-test using Prism 10 software. Primer sequences are listed in Supplemental Table 3.

**Western blot**

Cells were lysed in RIPA with Protease inhibitor cocktail (Roche). Total proteins were separated on SDS/PAGE gel and transferred to nitrocellulose membrane (Whatman). 5% non-fat milk in TBST were used for membrane blocking and antibody dilution. The membrane was incubated with primary antibody against HA-Tag (Proteintech, 51064-2-AP) at 4°C overnight. After twice TBST washing, the membrane was incubated with peroxidase-conjugated secondary antibodies (ZSGB-Bio). Bands were revealed by ECL reagent (Pierce).

**Supplementary Figure Legends**

**Fig S1. CHEK1 interactors enriched in GO term of cell cycle and DNA metabolic related processes**

1. PANTHER pathway analysis of CHEK1 interactors.
2. Gene ontology analysis of CHEK1 interactors by biological process (BP), molecular function (MF) and cellular component (CC).

**Fig S2. CHEK1 structure and overexpression in 293FT cells**

1. Domains and structures in the CHEK1 protein by Uniprot (<https://www.uniprot.org/>).
2. AlphaMissense Pathogenicity Heatmap for predicting the pathogenicity of missense mutations by Alphafold2. The x-axis represents the residue sequence number of amino acids in the protein, the y-axis shows alternative amino acids. The color gradient, ranging from blue (indicating low pathogenicity, close to 0) to red (indicating high pathogenicity, close to 1), depicts the pathogenicity score. The variant A26G is marked with a score of 0.754, located in a relatively red area, suggesting a potentially high pathogenicity.
3. Immunostaining showing the ectopic overexpression of WT and A26G CHEK1 in 293FT cells. Green, HA-Tag fused with WT or A26G CHEK; Red, F-Actin showing the cell shape; Blue, DAPI.
4. Dot plot illustrating the percentages of mitotic cells in negative control (NC), WT and A26G CHEK1 groups. Lines and columns are means ± SEM.
5. Representative images for cell nuclear stanning patterns during interphase and mitotic phase. Blue, DAPI.

**Fig S3. Comparison of transcriptome and splicing change between CHEK1 A26G and WT**

1. Venn diagram showing the number of differentially expressed genes (DEGs) between pairwise comparisons of the NC, WT and A26G CHEK1 overexpressed groups.
2. Heatmap of differentially expressed genes of WT and A26G CHEK1 overexpressed 293FT cells.
3. Volcano plot of differentially expressed genes between 293FT cells overexpressing WT and A26G CHEK1.
4. Scatter plot showing the statistics of KEGG pathway enrichment. Top 20 enriched pathways were shown.
5. Bar plot showing the gene ontology of DEGs between WT and A26G CHEK1 overexpressed cells by biological process (BP), molecular function (MF) and cellular component (CC).
6. Bar blot indicates IFIT1 and IFIT2 expression level of NC, WT and A26G CHEK1 overexpressed cells by FPKM.
7. Validation of IFIT1 and IFIT2 expression level of NC, WT and A26G CHEK1 overexpressed cells by quantitative-PCR.
8. Gene ontology analysis of alternative spliced genes between WT and A26G CHEK1 overexpressed 293FT cells by biological process, molecular function and cellular component.
