# Supplementary figures and images for: CHEK1 variant is a risk factor for premature ovarian insufficiency by mis- regulating metabolism and inflammation-related genes
Source: Hum Genomics. 2025 Jun 18;19:67. doi: 10.1186/s40246-025-00774-1 (PMC12178055; doi:10.1186/s40246-025-00774-1)

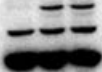

Supplement: Supplementary file 3 — Additional file 3. Supplementary Material. Raw Western Blotting. [file 40246_2025_774_MOESM3_ESM.pdf]
