# Supplementary material for: CHEK1 variant is a risk factor for premature ovarian insufficiency by mis- regulating metabolism and inflammation-related genes
Source: Hum Genomics. 2025 Jun 18;19:67. doi: 10.1186/s40246-025-00774-1 (PMC12178055; doi:10.1186/s40246-025-00774-1)
Supplement: Supplementary file 5 — Additional file 5. Supplementary Table 3. Varsome prediction. [file 40246_2025_774_MOESM5_ESM.pdf]

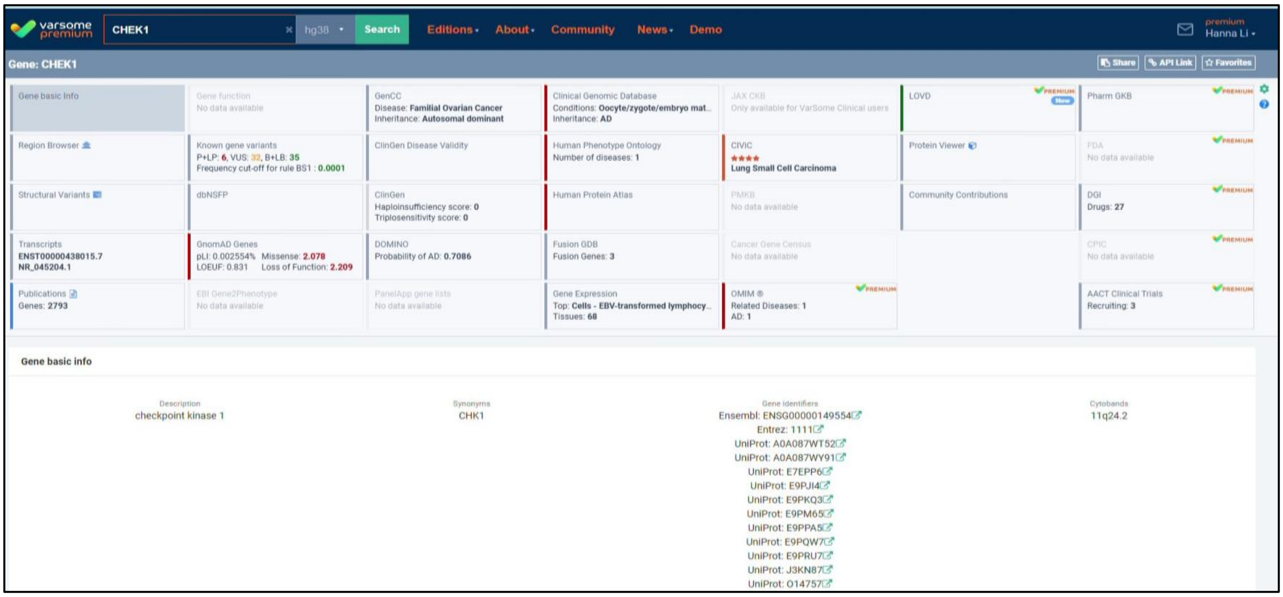
varsome premium
CHEK1
hg38
Search
Editions
About
Community
News
Demo
premium
Hanna Li
Gene: CHEK1
Share
API Link
Favorites
Gene basic Info
Gene function
No data available
GenCC
Disease: Familial Ovarian Cancer
Inheritance: Autosomal dominant
Clinical Genomic Database
Conditions: Oocyte/zygote/embryo mat...
Inheritance: AD
JAX CKB
Only available for VarSome Clinical users
LOVD
Pharm GKB
Region Browser
Known gene variants
P+LP: 6, VUS: 32, B+LB: 35
Frequency cut-off for rule BS1 : 0.0001
ClinGen Disease Validity
Human Phenotype Ontology
Number of diseases: 1
CIViC
Lung Small Cell Carcinoma
Protein Viewer
FDA
No data available
Structural Variants
dbNSFP
ClinGen
Haploinsufficiency score: 0
Triplosensitivity score: 0
Human Protein Atlas
PMKB
No data available
Community Contributions
DGI
Drugs: 27
Transcripts
ENST00000438015.7
NR_045204.1
GnomAD Genes
pLI: 0.002554% Missense: 2.078
LOEUF: 0.831 Loss of Function: 2.209
DOMINO
Probability of AD: 0.7086
Fusion GDB
Fusion Genes: 3
Cancer Gene Census
No data available
CPIC
No data available
Publications
Genes: 2793
EBI Gene2Phenotype
No data available
PanelApp gene lists
No data available
Gene Expression
Top: Cells - EBV-transformed lymphocy...
Tissues: 68
OMIM ®
Related Diseases: 1
AD: 1
AACT Clinical Trials
Recruiting: 3
Gene basic info
Description
checkpoint kinase 1
Synonyms
CHK1
Gene identifiers
Ensembl: ENSG00000149554
Entrez: 1111
UniProt: A0A087WT52
UniProt: A0A087WY91
UniProt: E7EPP6
UniProt: E9PJI4
UniProt: E9PKQ3
UniProt: E9PM65
UniProt: E9PPA5
UniProt: E9PQW7
UniProt: E9PRU7
UniProt: J3KN87
UniProt: O14757
Cytobands
11q24.2

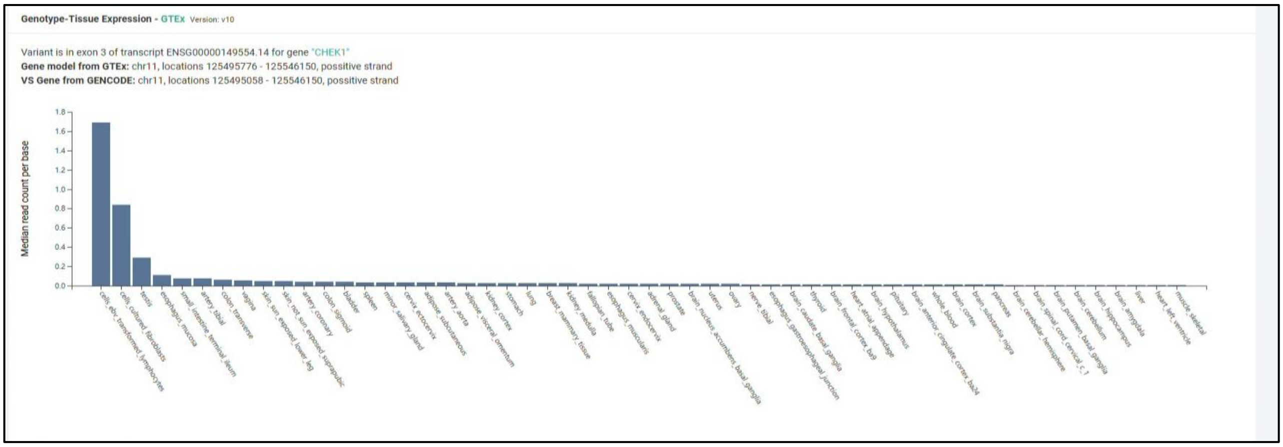
Genotype-Tissue Expression - GTEx Version: v10
Variant is in exon 3 of transcript ENSG00000149554.14 for gene "CHEK1"
Gene model from GTEx: chr11, locations 125495776 - 125546150, possitive strand
VS Gene from GENCODE: chr11, locations 125495058 - 125546150, possitive strand
Median read count per base
cells_ebv_transformed_lymphocytes
cells_cultured_fibroblasts
testis
esophagus_mucosa
small_intestine_terminal_ileum
artery_tibial
colon_transverse
vagina
skin_sun_exposed_lower_leg
skin_not_sun_exposed_suprapubic
artery_coronary
colon_sigmoid
bladder
spleen
minor_salivary_gland
cervix_ectocervix
adipose_subcutaneous
artery_aorta
adipose_visceral_omentum
kidney_cortex
stomach
lung
breast_mammary_tissue
kidney_medulla
fallopian_tube
esophagus_muscularis
cervix_endocervix
adrenal_gland
prostate
brain_nucleus_accumbens_basal_ganglia
uterus
ovary
nerve_tibial
esophagus_gastroesophageal_junction
brain_caudate_basal_ganglia
thyroid
brain_frontal_cortex_ba9
heart_atrial_appendage
brain_hypothalamus
pituitary
brain_anterior_cingulate_cortex_ba24
whole_blood
brain_cortex
brain_substantia_nigra
pancreas
brain_cerebellar_hemisphere
brain_spinal_cord_cervical_c-1
brain_putamen_basal_ganglia
brain_cerebellum
brain_hippocampus
brain_amygdala
liver
heart_left_ventricle
muscle_skeletal

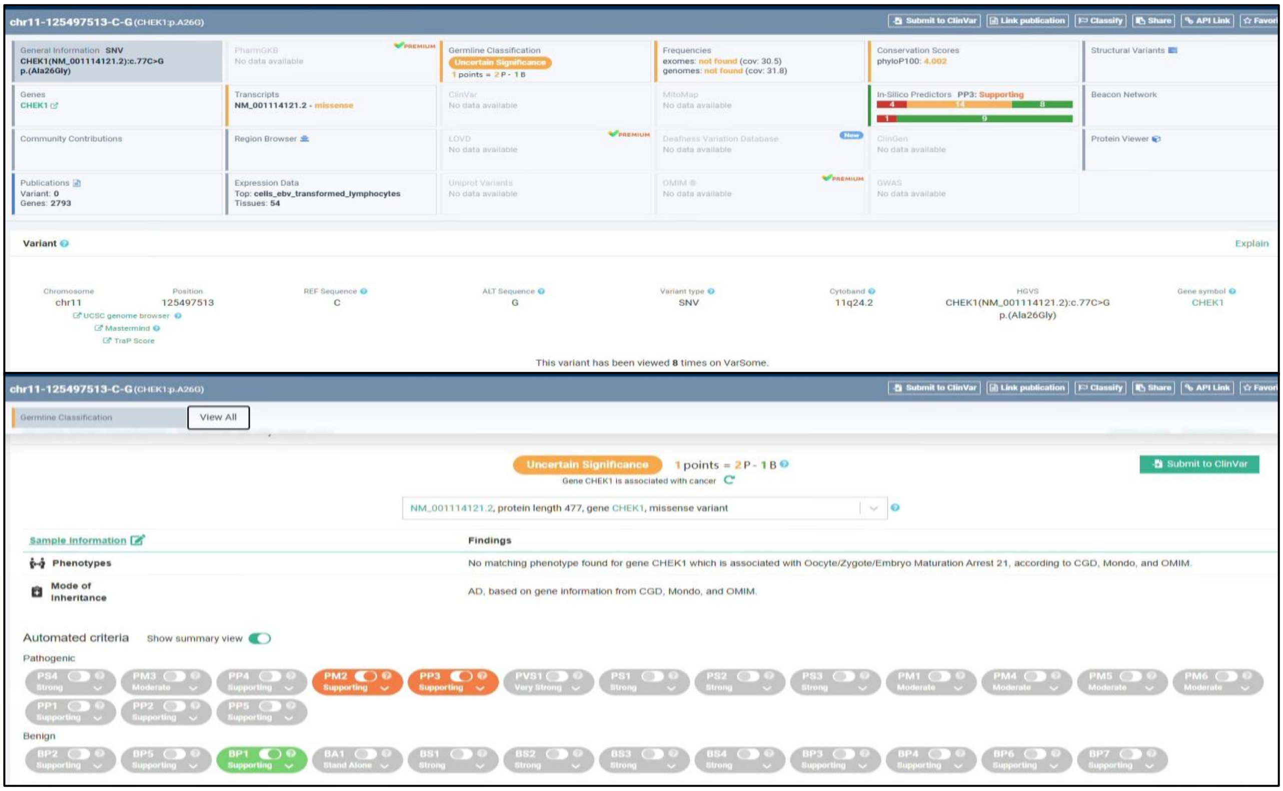
chr11-125497513-C-G (CHEK1:p.A26G)
Submit to ClinVar
Link publication
Classify
Share
API Link
General Information SNV
CHEK1(NM_001114121.2):c.77C>G
p.(Ala26Gly)
PharmGKB
No data available
Germline Classification
Uncertain Significance
1 points = 2 P - 1 B
Frequencies
exomes: not found (cov: 30.5)
genomes: not found (cov: 31.8)
Conservation Scores
phyloP100: 4.002
Structural Variants
Genes
CHEK1
Transcripts
NM_001114121.2 - missense
ClinVar
No data available
MitoMap
No data available
In-Silico Predictors PP3: Supporting
Beacon Network
Community Contributions
Region Browser
LOVD
Deafness Variation Database
No data available
ClinGen
No data available
Protein Viewer
Publications
Variant: 0
Genes: 2793
Expression Data
Top: cells_ebv_transformed_lymphocytes
Tissues: 54
Uniprot Variants
No data available
OMIM ®
No data available
GWAS
No data available
Variant
Explain
Chromosome
chr11
Position
125497513
REF Sequence
C
ALT Sequence
G
Variant type
SNV
Cytoband
11q24.2
HGVS
CHEK1(NM_001114121.2):c.77C>G
p.(Ala26Gly)
Gene symbol
CHEK1
UCSC genome browser
Mastermind
TraP Score
This variant has been viewed 8 times on VarSome.
Germline Classification
View All
Uncertain Significance
1 points = 2 P - 1 B
Gene CHEK1 is associated with cancer
Submit to ClinVar
NM_001114121.2, protein length 477, gene CHEK1, missense variant
Sample Information
Findings
Phenotypes
No matching phenotype found for gene CHEK1 which is associated with Oocyte/Zygote/Embryo Maturation Arrest 21, according to CGD, Mondo, and OMIM.
Mode of Inheritance
AD, based on gene information from CGD, Mondo, and OMIM.
Automated criteria
Show summary view
Pathogenic
PS4 Strong
PM3 Moderate
PP4 Supporting
PM2 Supporting
PP3 Supporting
PVS1 Very Strong
PS1 Strong
PS2 Strong
PS3 Strong
PM1 Moderate
PM4 Moderate
PM5 Moderate
PM6 Moderate
PP1 Supporting
PP2 Supporting
PP5 Supporting
Benign
BP2 Supporting
BP5 Supporting
BP1 Supporting
BA1 Stand Alone
BS1 Strong
BS2 Strong
BS3 Strong
BS4 Strong
BP3 Supporting
BP4 Supporting
BP6 Supporting
BP7 Supporting

In-Silico Predictors

Meta scores 1 | 9

Show raw data Alphabetically

Engine	Calibrated Prediction	Score	Indicative Prediction	Rankscore	Version
BayesDel noAF	Benign Moderate	noAF score<br>-0.2666	Tolerated	noAF rankscore<br>0.4816	dbNSFP version 4.9
MetaLR	Benign Moderate	0.1398	Tolerated	0.4588	dbNSFP version 4.9
MetaSVM	Benign Moderate	-0.9791	Tolerated	0.3521	dbNSFP version 4.9
REVEL	Benign Moderate	0.184, 0.184, 0.184, 0.184, 0.184, 0.184, 0.184, 0.184, 0.184		0.4576	dbNSFP version 4.9
MetaRNN	Pathogenic Supporting	0.8269, 0.8269, 0.8269, 0.8269, 0.8269, 0.8269, 0.8269, 0.8269, 0.8269, 0.8269	Damaging, Damaging, Damaging, Damaging, Damaging, Damaging, Damaging, Damaging, Damaging, Damaging	0.8187	dbNSFP version 4.9
BayesDel addAF	Benign Supporting	addAF score<br>-0.0201	Tolerated	addAF rankscore<br>0.4884	dbNSFP version 4.9

Individual Predictions 4 | 14 | 8

Show raw data Alphabetically

Engine	Calibrated Prediction	Score	Indicative Prediction	Rankscore	Version
MutPred	Pathogenic Strong	0.862, 0.862, 0.862, 0.862, 0.862, 0.862, 0.862, 0.862, 0.862, 0.862		0.9589	dbNSFP version 4.9
M-CAP	Benign Moderate	0.009196	Tolerated	0.2418	dbNSFP version 4.9
Mutation assessor	Benign Moderate	1.04, 1.04, 1.04, 1.04, 1.04	Low, Low, Low, Low, Low	0.2619	dbNSFP version 4.9
DEOGEN2	Benign Supporting	0.3115, 0.3115, 0.04659, 0.1291, 0.3115, 0.3115, 0.04843	Tolerated, Tolerated, Tolerated, Tolerated, Tolerated, Tolerated, Tolerated	0.6834	dbNSFP version 4.9
EIGEN	Benign Supporting	raw coding<br>0.004679		raw coding rankscore<br>0.4207	dbNSFP version 4.9
EIGEN PC	Benign Supporting	PC raw coding score<br>0.1834		PC raw coding rankscore<br>0.4894<br>PC phred coding score<br>3.1031	dbNSFP version 4.9
FATHMM	Benign Supporting	1.69, 1.69, 1.69, 1.69, 1.69, 0.53, 1.69, 1.69, 1.69	Tolerated, Tolerated, Tolerated, Tolerated, Tolerated, Tolerated, Tolerated, Tolerated, Tolerated	converted rankscore<br>0.5509	dbNSFP version 4.9
AlphaMissense	Uncertain	0.7542	Likely Pathogenic		version 03-Jul-2024
BLOSUM	Uncertain	-1			version BLOSUM100
CADD PREMIUM	Uncertain	24.7			version 1.7<br>Since version 1.6, CADD integrates several splice prediction scores, see publication
DANN	Uncertain	0.9966			version 2014
FATHMM-MKL	Uncertain	coding score<br>0.9303	Damaging	coding rankscore<br>0.5715	dbNSFP version 4.9
FATHMM-XF	Uncertain	coding score<br>0.7279	Damaging	coding rankscore<br>0.6761	dbNSFP version 4.9
LIST-S2	Uncertain	0.9659, 0.9681, 0.9588, 0.9654, 0.9659	Damaging, Damaging, Damaging, Damaging, Damaging	0.8841	dbNSFP version 4.9
LRT	Uncertain	0.000069	Deleterious	converted rankscore<br>0.5235<br>Omega<br>0.1307	dbNSFP version 4.9
MutationTaster	Uncertain	0.9999, 0.9999, 0.9999, 0.9999, 0.9999, 0.9999, 1	Disease causing, Disease causing, Disease causing, Disease causing, Disease causing, Disease causing, Disease causing	converted rankscore<br>0.81	dbNSFP version 4.9
MVP	Uncertain	0.8215, 0.8215, 0.8215, 0.8215, 0.8215, 0.8215, 0.8215, 0.8215, 0.8215, 0.8215		0.8198	dbNSFP version 4.9
PrimateAI	Uncertain	0.6479	Tolerated	0.5969	dbNSFP version 4.9
PROVEAN	Uncertain	-3.06, -3.06, -3.15, -3.3, -3.38, -3.43, -3.06, -3.06, -3.06	Damaging, Damaging, Damaging, Damaging, Damaging, Damaging, Damaging, Damaging, Damaging	converted rankscore<br>0.6736	dbNSFP version 4.9
SIFT	Uncertain	0.007, 0.007, 0.178, 0.122, 0.007, 0.057, 0.007, 0.007, 0.011	Damaging, Damaging, Tolerated, Tolerated, Damaging, Tolerated, Damaging, Damaging, Damaging	converted rankscore<br>0.5993	dbNSFP version 4.9
SIFT4G	Uncertain	0.079, 0.009, 0.009, 0.249, 0.27, 0.044, 0.032, 0.009, 0.009, 0.082	Tolerated, Damaging, Damaging, Tolerated, Tolerated, Damaging, Damaging, Damaging, Damaging, Tolerated	converted rankscore<br>0.6676	dbNSFP version 4.9
